# Effects of CFTR-modulator triple therapy on sinunasal symptoms in children and adults with cystic fibrosis

**DOI:** 10.1007/s00405-023-07859-4

**Published:** 2023-02-04

**Authors:** Sebastian F. N. Bode, Hannes Rapp, Nadine Lienert, Heike Appel, Dorit Fabricius

**Affiliations:** 1 Department of Pediatrics and Adolescent Medicine, Ulm University Medical Center, Ulm University, Eythstr 24, 89075 Ulm, Germany; 2 Department of Otorhinolaryngology, Head and Neck Surgery, Ulm University Medical Center, Ulm, Germany

**Keywords:** Cystic fibrosis, Sinunasal symptoms, SNOT-22, Highly effective CFTR-modulator therapy

## Abstract

**Purpose:**

Sinunasal symptoms and chronic rhinusinutitis are common in patients with cystic fibrosis. Cystic fibrosis transmembrane regulator (CFTR) modulators have led to dramatic improvements of respiratory symptoms and quality of life in patients with cystic fibrosis. This study aims to evaluate subjective and objective sinunasal symptoms after start of CFTR-modulator triple therapy.

**Methods:**

43 patients (*n* = 6 < 18 years), treated with highly effective CFTR-modulator therapy with elexacaftor–tezacaftor–ivacaftor (ELX/TEZ/IVA) were included, as were 20 controls with cystic fibrosis but without CFTR-modulator therapy (*n* = 6 < 18 years). All assessed their sinunasal symptoms retrospectively and the intervention group at a mean of 9.3 (2–16) months after start of ELX/TEZ/IVA.

**Results:**

Improvements in SNOT-22 overall score from *m* = 32.7 to *m* = 15.7 points (*p* < 0.0001) as well in the nasal, emotional, otologic, and sleep subdomains could be demonstrated in the intervention group. No changes were found in the control group. Children showed lower SNOT-22 scores than adults and a reduction of SNOT-22 total score from *m* = 9.4 to *m* = 2.2 (*p* = 0.25) was found. 8 patients were evaluated by an otorhinolaryngologist before and after start of ELX/TEZ/IVA and showed pronounced objective clinical improvement.

**Conclusions:**

Highly effective CFTR-modulator therapy has a significant positive impact on both subjective and objective sinunasal symptoms in patients with CF and some improvement could be demonstrated in children < 18 years as well.

## Background

Cystic fibrosis (CF) is characterized by mutations in the CF transmembrane regulator (*CFTR*) gene. The CFTR-protein is a chloride and bicarbonate channel and mutations cause impaired transport of ions through epithelial barriers leading to pathological mucus viscosity. Pulmonary disease, upper respiratory tract dysfunction, exocrine pancreatic insufficiency, as well as disorders of gastrointestinal and reproductive systems are part of the clinical picture of CF. In the upper airways, reduced mucociliary clearance and hyperviscous mucus lead to mucus retention and chronic inflammation, causing chronic rhinosinusitis (CRS) [1–3]. Symptoms congruent with CRS are found in almost all patients with CF in imaging studies or on endoscopic evaluation but only 10–15% of adults and 20% of children report CRS symptoms [1–3]. Up to two thirds of CRS-symptomatic CF patients have had some form of otorhinolaryngology intervention [4]. Most patients though report to be asymptomatic. Many patients experience sinunasal dysfunction since birth or early childhood and therefore adapt to pathological conditions. Additionally, pulmonary, gastro-intestinal, or endocrine problems take precedence in many patients and sinunasal symptoms are perceived secondary to other clinical problems or less burdensome [5, 6].

Therapeutic options for CRS in CF are limited and a recent Cochrane review found a lack of evidence regarding therapy [7]. There is some evidence for beneficial effects of nasal saline, endoscopic sinus surgery, and intranasal corticosteroids as well as topical antibiotics [1, 8]. CFTR-modulator therapy has been shown to improve lung function, exacerbation rate, body mass index, quality of life and other aspects of life of patients with cystic fibrosis [9, 10]. Effects of CFTR-modulator therapy have been shown in CF-CRS. Case reports and small case series show that therapy with ivacaftor, approved for treatment of CF patients with G551D mutations, can improve sinunasal symptoms as well as radiological signs of CRS [11–13]. Positive effects of ivacaftor regarding quality of life, psychological and sleep domains, evaluated with the 22 item sinu-nasal outcome test (SNOT-22) questionnaire, have been reported [14]. Combining the CFTR-potentiator ivacaftor with the CFTR-corrector lumacaftor has shown positive results on lung function, growth, and reduced the risk of pulmonary exacerbations [15] but no effects on CRS have been reported. For tezacaftor, another CFTR-corrector, in combination with ivacaftor, no data on changes in sinunasal symptoms have been reported. Triple therapy with ivacaftor and the correctors tezacaftor as well as elexacaftor (ELX/TEZ/IVA) has shown dramatic improvement of pulmonary function (forced expiratory volume in 1 s, FEV-1), quality of life, body mass index, and reduction of exacerbations and has been termed highly effective CFTR-modulator therapy [10, 16]. Functional data demonstrate significant improvement of CFTR-function in nasal epithelia after start of ELX/TEZ/IVA [17]. Significant improvement of sinunasal and respiratory symptoms as well as quality of life were reported for CFTR-modulator triple therapy in adults [18–21]. Radiologic imaging showed positive changes in paranasal sinus abnormalities in adults after start of highly effective modulator therapy [21, 22].

We sought to correlate subjective changes, measured by the validated 22-item sinu nasal outcome test [23]), with objective changes in sinunasal symptoms, and clinical findings in children and adults with CF who initiated highly effective CFTR-modulator therapy. The SNOT-22 has been routinely used to assess CRS symptoms in CF-patients [18, 19] and the German version has been validated [24]. We used the validated four-factor subscale model with the subdomains ‘nasal symptoms’, ‘otologic symptoms’, ‘sleep symptoms’, and ‘emotional symptoms’ [25]. At the time of the study ELX/TEZ/IVA was approved for patients older than 12 years with at least one copy of F508del.

## Methods

We conducted a single-center, cross sectional study. All CF-patients of our outpatient clinic were offered participation in the study during a routine follow-up visit in August and September, 2021. Only patients not on other CFTR-modulator therapy were included and patients with organ transplants were excluded. Patients were asked to first retrospectively rate their sinunasal symptoms before starting ELX/TEZ/IVA therapy using the SNOT-22 questionnaire (timepoint 1, T1). Patients were then asked to rate their current symptoms after start of ELX/TEZ/IVA with a second set of the questionnaire (timepoint 2, T2). Patients who had not started ELX/TEZ/IVA, or were not eligible for ELX/TEZ/IVA, were included as controls and only rated their current symptoms with the SNOT-22. Clinical data (lung function, sweat chloride, nasal endoscopy) were collected. ELX/TEZ/IVA was initiated in the intervention group between timepoints. Questionnaires were filled out in paper–pencil format and data were then transferred into an electronic database. Both overall score and score of the subdomains of the SNOT-22 were appreciated. All patients were offered a clinical examination by an experienced otorhinolaryngologist for objective evaluation and nasal endoscopy after start of highly effective CFTR-modulator therapy. All examinations were performed by the same otorhinolaryngologist.

### Ethics

Ethics approval was obtained from the Ulm University ethics committee (permit no.: 228/21) and the study was registered in the German Registry for Clinical Studies (DRKS registry study ID 00025839) prior to inclusion of the first patient. Participants or, in case of participating children < 18 years, their parents, gave written informed consent to participate in the study.

### Data analysis and statistics

Statistical analysis was performed with GraphPad Prism (Version 7.01, GraphPad Software, La Jolla, CA, USA, www.graphpad.com). Categorial data are reported as frequencies, means, standard deviation, median, minimum, maximum, and quartiles. Mann–Whitney *U* tests/*T* tests for parallel groups were used to determine differences between the intervention and the control group. For temporal differences in the intervention group *T* tests for pairwise differences and sign rank tests were used, depending on the data distribution. Significance level was set at *α* < 0.05.

## Results

43 patients who had started ELX/TEZ/IVA therapy participated in the study as did 20 controls, who did not receive CFTR-modulator therapy. All participants received inhalative therapy, enzyme replacement, physiotherapy, etc. as recommended. Controls had a mean age of 18 years (30% children < 18 years) and patients in the intervention group had a mean age of 32 years (14% children < 18 years). There were more females in the intervention group. More patients in the intervention group had a homozygous F508del CFTR mutation and the intervention group had a lower FEV-1 before initiation of ELX/TEZ/IVA. Detailed demographic data can be found in Table 1. All participants in the intervention group retrospectively rated their sinunasal symptoms before start of ELX/TEZ/IVA and after a mean of 9.3 (2–16) months of therapy. Both FEV-1 increased significantly (*p* < 0.0001) and sweat chloride fell significantly (*p* < 0.0001) in the intervention group. Changes were significant in both children and adults (*p* < 0.01 for both FEV-1 and sweat chloride). Controls showed no changes over a similar time period.

**Table 1:** Demographic data of study participants

	Controls	Timepoint 1	Timepoint 2
		ELX/TEZ/IVA	ELX/TEZ/IVA
Number	20	43	43
Age, years (min–max)	18 (6–46)	32 (11–56)	33 (12–58)
Children < 18 years, *n* (%)	6 (30)	6 (14)	4 (9.3)
Females, *n* (%)	9 (45)	25 (58.1)	25 (58.1)
F508del homozygous, *n* (%)	6 (30)	23 (53,5)	23 (53,5)
FEV-1%predicted (± SD)	79.5 (18.5)	62.7 (22.9)	76.2 (26)
Sweat chloride, (± SD)	92 (11.4)	98.3 (16.9)	53.7 (20.7)

*n* number, *SD* standard deviation

**Fig. 1:**
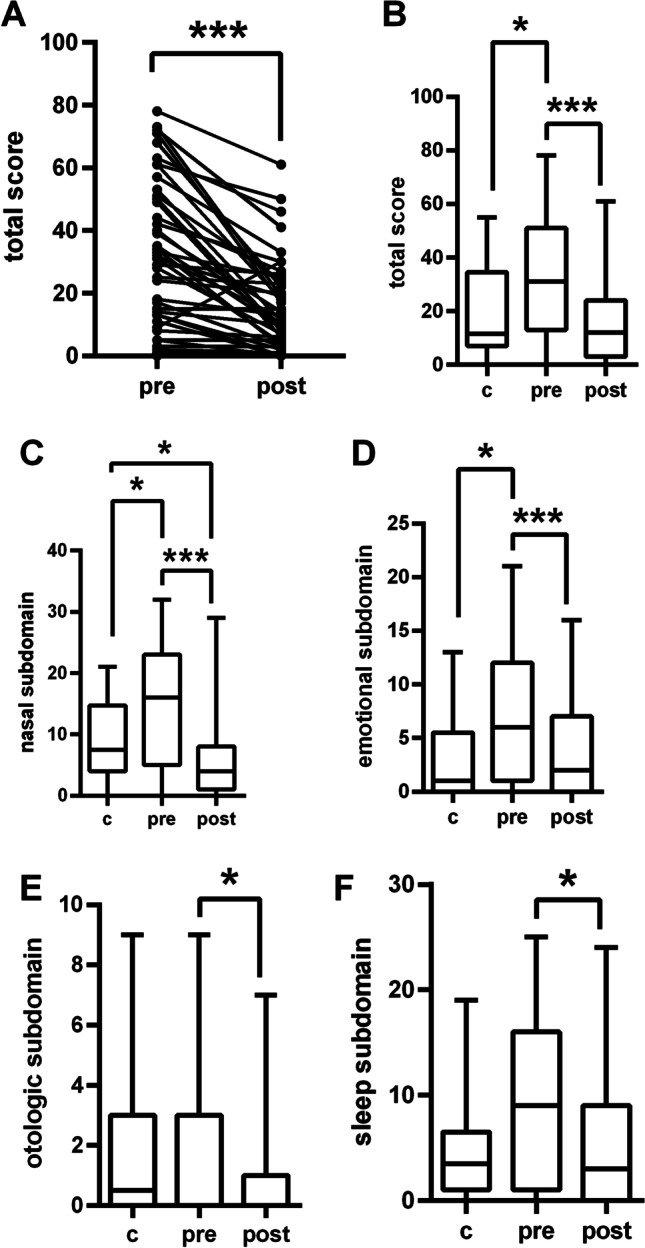
Improved sinu-nasal outcome test-22 (SNOT-22) after (post) 9.3 (2–16) months of therapy in 43 patients treated with ELX/TEZ/IVA compared to before (pre) therapy initiation and 20 controls (c). **A** Individual courses of total SNOT-22 score in the intervention group. **B** Total SNOT-22 score. **C** Nasal subdomain score of SNOT-22. **D** Emotional subdomain. **E** Otologic subdomain score of SNOT-22. **F** Sleep subdomain score of SNOT-22. C = controls, *p < 0.05, **p < 0.01; ***p < 0.001

Regarding sinunasal symptoms both overall score of the SNOT-22 as well as the four subdomains were evaluated as was the single olfactory question. Before initiation of highly effective CFTR-modulator therapy participants rated their total SNOT-22 score as well as their nasal and emotional subdomains worse than did controls. Total SNOT-22 score decreased by 17 points (*p* < 0.0001) after a median treatment duration of 9.3 (2–16) months (Table 2, Fig. 1A, B). Nasal, otologic, sleep, and emotional subdomains showed significant reductions of self-perceived symptoms in the intervention group (Fig. 1C–F). The nasal subdomain was even rated significantly better in the intervention group than in the control group after start of highly effective CFTR-modulator therapy (Table 2, Fig. 1C). One participant showed a significant increase within her total score. However, this could be explained by a stressful situation in the home environment, which resulted in higher scores in the emotional and sleep subdomains in this individual.

**Table 2:** Significant changes in SNOT-22 score before (T1) and 9.3 (2–16) months after (T2) starting ELX/TEZ/IVA therapy (*n* = 43) and in the context of a control group [*n* = 20; assessed at one time point (T2)]

SNOT-22	Mean (minimum–maximum)			Comparisons		
	Control group	T1 ELX/TEZ/IVA	T2 ELX/TEZ/IVA	T1/T2: *p* value (delta)	control/T1; *p* value (delta)	control/T2; *p* value (delta)
Total score	18.8 (0–55)	32.7 (0–78)	15.7 (0–61)	**< 0.0001 (− 17)**	**0.0336 (+ 13.9)**	0.42 (− 3.1)
Nasal subdomain	9.2 (0–21)	15.0 (0–32)	5.7 (0–29)	**< 0.0001 (− 9.3)**	**0.028 (+ 5.8)**	**0.026 (− 3.5)**
Emotional subdomain	4.2 (0–13)	7.1 (0–21)	3.6 (0–16)	**< 0.0001 (− 3.5)**	**0.01 (+ 2.9)**	0.54 (− 0.6)
Otologic subdomain	1.8 (0–9)	1.6 (0–9)	1.1 (0–7)	**0.024 (− 0.5)**	0.73 (− 0.2)	0.3 (− 0.7)
Sleep subdomain	5.2 (0–19)	9.0 (0–25)	5.3 (0–24)	**0.006 (− 3.7)**	0.081 (+ 3.8)	0.87 (+ 0.1)
olfaction	0.65 (0–5)	1.35 (0–5)	0.95 (0–5)	0.39 (**− **0.39)	0.28 (+ 0.69)	0.71 (+ 0.3)

Significant differences with a *p* < .05 are marked in bold

The nasal subdomain showed significant differences between the intervention and the control group both before and after starting the therapy with ELX/TEZ/IVA. Before start, the control group reported a score of 5.8 points less than the intervention group (*p* = 0.028). After 9.3 (2–16) months of therapy patients treated with ELX/TEZ/IVA reported significantly fewer nasal symptoms than the control group (*p* = 0.026) and compared to before initiation of therapy (*p* < 0.0001).

The otologic as well as the nasal, and the emotional subdomains showed significant reductions (*p* = 0.024, *p* = 0.006, and *p* ≤ 0.001, respectively) after start of highly effective CFTR-modulator therapy. The emotional subdomain was rated worse in the intervention group before treatment initiation compared to the control group. After start of highly effective modulator therapy no differences between intervention and control groups were found any more (Fig. 1).

The single olfactory question was rated similar in the control group as well as the intervention group before and after initiation of ELX/TEZ/IVA. Even though the control group showed slight improvement from *m* = 1.35 to *m* = 0.95 points this was not significant. The control subjects rated their olfaction with *m* = 0.65 and no significant differences to the intervention group were found (Table 2).

Total SNOT-22 scores were significantly lower in children than adults in both intervention and control groups at all timepoints (Table 3). SNOT-22 total score decreased in children after highly effective modulator therapy was initiated, but the changes were not significant (Table 3).

**Table 3:** SNOT-22 total score in children < 18 years and adults. Significantly lower total scores in children at all timepoints than in adults

	Control group	T1 ELX/TEZ/IVA	T2 ELX/TEZ/IVA
Children < 18 years: SNOT-22 total score	5.0 (0–19)	9.4 (1–31)	2.2 (0–6)
Adults: SNOT-22 total score	20.7 (4–55)	36.5 (0–78)	17.4 (0–61)
Comparisons children vs. Adults. *p* value (delta)	**0.003 (− 15.7)**	**< 0.001 (− 27.2)**	**< 0.001 (− 15.2)**
Comparison children pre/post. *p* value (delta)			0 .25 (− 7.1)

*ELX/TEZ/IVA* elexacaftor/tezacaftor/ivacaftor, *T* timepoint

Significant differences with a *p* < .05 are marked in bold

### Objective otorhinolaryngology findings

Eight patients of the intervention group opted for voluntary otorhinolaryngeal examination 9.3 (2–16) months after start of ELX/TEZ/IVA (Table 4) but none of the controls did. All patients seen in the otorhinolaryngeal department suffered from CRS. Five patients suffered from CRS with nasal polyps (CRSwNP) and three patients had CRS without nasal polyps. 6/8 patients had had a surgical intervention before, two patients had four previous surgeries each. Overall SNOT-22 score improved from *m* = 38.8 (12–63) points to *m* = 23.6 (2–50) points 10 (5–16) months after therapy onset. Seven patients reported improved sinunasal symptoms. Four patients reported improved ability to smell. One patient (Patient 5, Table 4) suffered from allergic rhinitis at the time of the examination and this individual showed no improvement in SNOT-22 score but improvement on examination. On endoscopic evaluation clinical improvements were documented in five patients (Table 4).

**Table 4:** Relevant findings in otorhinolaryngeal follow-up in 8 patients before and after start of ELX/TEZ/IVA-therapy

Patient no (age, sex)	Duration of therapy	SNOT-22		Previous nasal surgeries, no	Symptoms		Nasal endoscopy	
		T1	T2		T1	T2	T1	T2
1 (43, m)	16	49	6	1	Nasal airway obstruction, hyposmia	No symptoms	Nasal polyps on the left side, mucosal swelling on the right side	Minimal polyp on the left side
2 (51, f)	10	51	24	1	Rhinorrhea, sinusitis	Improvement of symptoms	Normal	Normal
3 (46, f)	7	32	12	4	Nasal airway obstruction, weekly nasal suctioning, cephalgias, hyposmia	No nasal obstruction, no nasal suctioning, no cephalgias, hyposmia	Nasal polyps both sides, strong nasal borking	No polyps, mild scarring
4 (29, f)	10	61	50	No	Rhinorrhea	Rhinorrhea	Normal	Normal
5 (26, m)	5	12	23	1	Nasal airway obstruction, anosmia	No obstruction, hyposmia	NPS 3–4	No polyps, mild mucous erythema
6 (32, f)	9	13	2	No	Sinusitis	Improvement of symptoms	Normal	Normal
7 (36, f)	10	63	46	2	Nasal airway obstruction, anosmia	No obstruction, normosmia	NPS 4	NPS 1
8 (39, f)	13	29	26	4	Nasal airway obstruction, cephalgias, hyposmia	No obstruction, no cephalgias, normosmia	NPS 1	No polyps

*ELX/TEZ/IVA* ivacaftor/tezacaftor/elexacaftor, *f* female, *m* male, *NPS* nasal polyps score, *no* number

## Discussion

The cohort of CF-patients presented here have typical initial FEV-1 and sweat chloride values as well as BMI as expected of CFTR-modulator naïve CF-patients. The intervention group included older patients with more advanced disease compared to the control group. All parameters changed after initiation of highly effective modulator therapy in the intervention group as expected and changes are congruent with previous results [9, 10]. No changes were found in the control group who were not treated with CFTR-modulators. The intervention group showed significantly worse overall SNOT-22 scores as well as worse scores in nasal and emotional subdomains compared to the control group prior to therapy. We could demonstrate a significant improvement of the SNOT-22 total score with an average reduction of 17 points 9.3 months after initiation of highly effective modulator therapy (*p* < 0.001), greater than the minimally clinically significant difference for the SNOT-22 [23]. In addition, significant improvements were seen in all subdomains. The nasal subdomain improved by 9.3 points (*p* < 0.001), the emotional subdomain by 3.5 points (*p* < 0.001), the otologic subdomain by 0.5 points (*p* = 0.024), and the sleep subdomain by 3.7 points (*p* = 0.006). After start of the therapy the intervention group reported similar total and subdomain scores as the control group and even an improved nasal subdomain score. No significant changes were found regarding olfaction.

DiMango et al. reported an improved SNOT-22 score by 10.4 points as well as improvements in all subdomains three months after initiation of highly effective modulator therapy [18]. A reduction of 10.2 points five months after initiation of ELX/TEZ/IVA as well as improvements in all but the sleep and psychological subdomains were reported in a cohort of 25 adults with CF, who all had a history of previous otorhinolaryngological surgery [19] and similar results have been reported in two cohorts of 32 and 25 adults patients with advanced CF 6 months after therapy onset [20, 21]. Olfaction seems to be problematic even after start of highly effective CFTR-modulator therapy [26]. Even though we found a discreet trend in improvement in the one olfactory question of the SNOT-22 and in some of the subjects evaluated by an otorhinolaryngologist this was not significant.

Our data are in accordance with the published literature but offer insights into treatment success on CF-CRS with highly effective CFTR-modulator therapy in a longer timeframe of 9.3 months. As the reduction of the SNOT-22 score in our cohort was larger than in other cohorts reported before, longer treatment might have a more pronounced effect on sinunasal symptoms. Additionally, a small subcohort of children < 18 years of age was analyzed. To our knowledge these data are the first that demonstrate a positive effect of highly effective modulator therapy on CRS symptoms in children with CF. Additionally we could demonstrate objective improvements in nasal congestion and polyp size on clinical examination. While only examined in a small sub-cohort, this objective assessment supports the positive effects of the CFTR-modulator therapy on CRS symptoms. Our data support the statement of the Cystic Fibrosis Foundation that highly effective modulator therapy should be offered to all CF patients with appropriate age and genotype who suffer from CRS, and might even be offered in the absence of pulmonary symptoms in these patients [27].

### Strengths and limitations

The major strength of the study is that it takes both patients’ subjective symptoms as well as objective symptoms of a subcohort on otorhinolaryngeal examination into account. The follow-up of more than 9 months after initiation of highly effective CFTR-modulator therapy is longer than previous timeframes reported. To our knowledge no data on pediatric patients with CF and CRS, who were treated with highly effective modulator therapy, have been reported before. Limitations include the small sample size and the analysis of different subgroups, so results should not be generalized to the whole CF population. As patients rated their CRS symptoms before start of ELX/TEZ/IVA retrospectively their appraisal of symptoms might be biased. As the start of ELX/TEZ/IVA has a significant impact on clinical symptoms and patients recall symptoms before CFTR-modulator therapy quite clearly, we think this retrospective evaluation is still beneficial. Unfortunately the control group only rated their symptoms at one time-point and no control subjects participated in the objective otolaryngeal examination—so comparisons between the two groups in that regard are not possible.

## Conclusion

Highly effective CFTR-modulator therapy has substantial benefits for CF patients regarding otorhinolaryngeal symptoms. In our cohort a reduction of symptoms in both subjective assessment with SNOT-22 overall score as well as in all four subdomains and improvement on objective otorhinolaryngology examination could be demonstrated. In a small pediatric subcohort positive changes in SNOT-22 overall score could also be demonstrated but did not reach statistical significance. This study adds to the evidence that highly effective CFTR-modulator therapy has benefits on upper airway symptoms in CF patients.

## Data Availability

Data are available upon reasonable request from the corresponding author.
